# A Novel DEM Block Adjustment Method for Spaceborne InSAR Using Constraint Slices

**DOI:** 10.3390/s22083075

**Published:** 2022-04-16

**Authors:** Rui Wang, Huiming Chai, Bin Guo, Li Zhang, Xiaolei Lv

**Affiliations:** 1 Key Laboratory of Technology in Geo-Spatial Information Processing and Application System, Chinese Academy of Sciences, Beijing 100090, China; wangrui185@mails.ucas.ac.cn (R.W.); chaihm@aircas.ac.cn (H.C.); zhangli174@mails.ucas.ac.cn (L.Z.); 2 Aerospace Information Research Institute, Chinese Academy of Sciences, Beijing 100094, China; 3 School of Electronic, Electrical and Communication Engineering, University of Chinese Academy of Sciences, Beijing 100049, China; 4 Beijing Capital International Airport Group, Beijing Daxing International Airport, Beijing 102604, China; guo.b@bdia.com.cn

**Keywords:** constraint slice (CS), constraint conditions, DEM block adjustment, Interferometric Synthetic Aperture Radar (InSAR), matrix perturbation theory

## Abstract

The lack and uneven distribution of Ground Control Points (GCPs) will lead to the deterioration of Digital Elevation Model (DEM) block adjustment results in the bistatic Interferometric Synthetic Aperture Radar (InSAR) system. Given this issue, we first explain the relationship between the stability of adjustment parameters and the GCP distribution pattern theoretically using matrix perturbation theory. Second, we put forward the Constraint Slices (CSs) concept and first introduce CSs into the adjustment optimization model as constraint conditions rather than actual values as GCPs. Finally, we propose a novel DEM block adjustment method for spaceborne InSAR using CSs based on an optimization model with nonlinear constraints. The simulated experiment shows the instability of the conventional method and validates the proposed method under different parallel baseline errors. Four groups of real experiments were carried out according to the size of the uncontrolled area using twelve Co-registered Single-look Slant–range Complex (CoSSC) datasets for Henan Province, China. The adjustment results verified by the ICESat-2 ATL08 data demonstrate that the performance of the proposed method is better than the conventional method in the uncontrolled area; the corresponding improvements in adjustment accuracies compared with the conventional method are 0.13 m, 1.02 m, 2.12 m, and 8.18 m, respectively. At the same time, the proposed method can enhance the height consistency in overlapping areas, which is vital for seamless DEM production.

## 1. Introduction

The Bistatic Interferometric Synthetic Aperture Radar (InSAR) system [1] can effectively suppress the phase components caused by atmospheric delay and other factors to maintain the high coherence of interferograms, which is the leading technology to generate high-precision global DEM. TerraSAR-X/TanDEM-X is a typical example of the bistatic system, and has a baseline accuracy of about 1–2 mm [2,3].

The generation of interferometric DEM usually includes SAR image coregistration, interferometric processing, interferogram filtering, removing reference phases, phase unwrapping, elevation inversion, and geocoding [1,4]. The evaluation indicators of generated DEM include vertical accuracy and plane accuracy. Vertical accuracy is principally affected by the uncertainty of the spatial baseline, while plane accuracy is limited to the uncertainty of the parameters related to SAR imagery geolocation. Referring to the method of SAR bundle adjustment, J.Ma et al. [5] has proposed the Range-Doppler-Phase (RDP) model to correct key geometric parameters used in the R-D model and DEM inversion, including near range, imaging start time, orbit vector, and baseline. However, in recent years the geolocation accuracy of SAR satellites has improved from the hundreds of meters [6] to the decimeter order [7] with the development of satellite orbit determination and SAR calibration technology. For example, the orbital accuracy of TerraSAR-X is 20 cm, and the positioning accuracy can reach the order of decimeters [8,9]. Therefore, it is unnecessary to adjust geolocation parameters for spaceborne SAR systems with high positioning accuracy.

Because the SAR instrument calibration and the baseline determination cannot reduce all errors, there is a height error in the interferometric DEM caused by baseline uncertainty, instrument drifts, and other factors called the remaining systematic error [10]. Compared with the other factors, the parallel baseline error has a more significant impact on the accuracy of DEM generation, as follows:

(1) $$h_{err}=\frac{R\sin\theta }{B_{⊥}}B_{\|err}$$

where *R* is the slant range, θ is the incidence angle, $B_{⊥}$ is the perpendicular baseline, and $B_{\|err}$ is the parallel baseline error. In order to correct the remaining systematic error, B.Wessel et al. put forward a method of DEM calibration to correct the systematic error of DEM products [11], which was the core principle of the Mosaicking and Calibration Processor (MCP) of TanDEM-X [12]. In this method, the systematic error of the generated DEM is expressed as a polynomial function of the image coordinates and the function model is solved employing the external high-precision Ground Control Points (GCPs), that is, the Global Land Surface Altimetry Data (GLAH14) of the Ice, Cloud, and land Elevation Satellite (ICESat) [13]. J. Hueso Gonz$\overset{´}{a}$lez et al. [14] focused on the quality assessment of ICESat GLAH14 elevation data and put forward basic and extreme selection criteria according to the number of echo peaks and bandwidth of measurement points. The characteristics of the systematic error were analyzed in [15], and the simulation data were used to illustrate the necessity and reliability of DEM calibration. Astrid Gruber et al. [10] described the concept of the Tie-Point chip and the selection of ICESat data, as well as validating the function model using external lidar DEM.

GCPs are usually required to be sufficiently and evenly distributed to ensure the accuracy of DEM adjustment parameters [5,16,17]. Nevertheless, the GLAH14 data points are distributed along the ICESat orbit, which cannot meet these two requirements, especially the latter (the global distribution of ICESat data is shown on https://search.earthdata.nasa.gov/search?fp=ICESat&as[platform][0]=ICESat, accessed on 21 February 2022). Therefore, the adjustment results will deteriorate in certain cases and it is necessary to introduce external control data to ensure the accuracy of DEM adjustment parameters. However, this topic has seldom been discussed in previous studies on InSAR-DEM adjustment. The global public DEM can be regarded as reliable external control data. As an essential data source in geoscience, the global public DEM has high precision and wide coverage characteristics. The current global DEM/DSM can be divided into two categories according to the production technology; one is generated based on electromagnetic interference, such as Shuttle Radar Topography Mission (SRTM) [18] and the other is based on optical stereo pairs [19] such as ALOS World 3D (AW3D30) and the Advanced Spaceborne Thermal Emission and Reflection Radiometer Global Digital Elevation Model (ASTER GDEM). In addition, the National Elevation Dataset project has provided LiDAR DEM covering almost the whole of the United States [20]. Many scholars have evaluated the accuracy of global public DEM/DSM. R.Talchabhadel et al. [21] used thousands of ground-based points near the basin of the West Rapti River, Nepal, to access the vertical accuracy of AW3D30 and SRTM. The AW3D30 showed the highest accuracy of 3.41 m in the lower region of the study area. H.Li et al. [22] used ICESat data, SRTM, and ASTER GDEM to evaluate AW3D30 comprehensively. Their results showed that the horizontal offset of AW3D30 is approximately 5.0 m and 10.0 m, respectively, relative to SRTM and ASTER GDEM2, while it has a higher vertical accuracy of 4.81 m than the others. However, it should be noted that the vertical accuracy of public DEM is generally less than that of GLAH14 laser altimetry data, which means that the public DEM cannot be directly regarded as GCPs in adjustment as otherwise it will affect the correction effect of GLAH14 data on the generated DEMs. Therefore, it is necessary to develop an appropriate form to introduce the global public DEM into DEM block adjustment in order to solve the deterioration caused by the lack or uneven distribution of GCPs.

Based on the above analysis, this paper first analyzes the influence of ICESat distribution on adjustment results using matrix perturbation theory. Then, we propose the concept of constraint slices (CS) and the corresponding extraction method from external public DEM. Finally, taking the relative accuracy of CSs as constraint conditions, we propose a DEM block adjustment method assisted by CSs. A simulated experiment was designed according to different spatial baseline errors, and the overall adjustment accuracies of the simulated experiment improved by 0.41 m to 7.01 m. Four groups of real data experiments were designed according to the number of GCPs, and the overall adjustment accuracies improved by 0.02 m to 7.86 m.

## 2. Methods

### 2.1. Conventional Method and Shortcomings

The conventional method [10,11,14] models the remaining systematic height error of InSAR DEMs as a polynomial function and uses Tie Point (TP) chips and GCPs for joint correction.

#### 2.1.1. TP Chips

**Figure 1:**
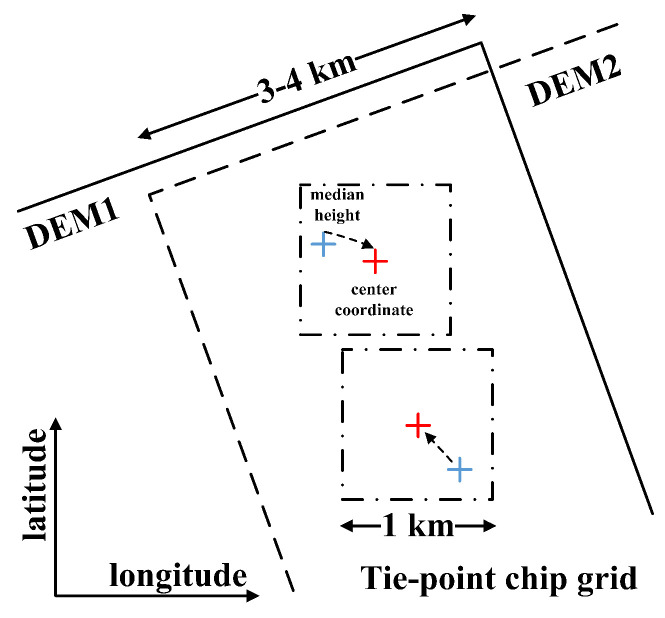
Extraction of TP chips. The dashed box indicates the tie point chip, the red mark is the center point, and the blue mark is the location of the elevation median.

In the overlapping area of two DEMs, pixels corresponding to the same ground object are called tie points [11]. This concept is similar to the connections/tie points in SAR imagery block adjustment [23], though their extraction methods are different. Gaussian white noise usually contaminates the generated DEM, and the pixels near the edge may even be affected by colored noise [11]. To ensure the relative accuracy and reliability of connection points, Huber M. et al. put forward the concept of TP chips and developed a set of extraction processes [24]. First, the image coherence threshold is set to eliminate the pixels with low coherence. At the same time, the areas with higher height differences compared with the public external DEM are removed to eliminate the invalid areas, such as shadow, water, and overlap areas. Then, a series of grids called TP chips are delimited along the longitude and latitude direction in the overlapping area. A median height value is assigned to a TP chip after calculating the histogram of effective pixels in each grid. The geographic coordinate of a TP chip is that of the center point in the grid. Compared with feature matching, this method is more time-saving [10,11,24]. It is able to realize time savings of more than 95.0% thanks to the extraction of TP chips mainly using logical operations such as establishing grids and masking invalid areas. The extraction diagram of TPs is shown in Figure 1.

#### 2.1.2. GCPs

ICESat data provide globally distributed accurate elevation measuring points, and has been used as an absolute height reference for DEM adjustment [10,11]. ICESat data are measured with lasers, which means the measurement points are affected by clouds, vegetation, and other factors and cannot reflect the actual ground elevation. Therefore, J.H. González et al. [14] focused on ICESat data screening in DEM adjustment and put forward extreme and basic selection criteria. The specific process is as follows. First, the ICESat points with significant height differences (greater than 200 m) are eliminated through comparison with external DEM/DSM. Then, data points with one peak for the reflected signal and a time bandwidth of fewer than 3.2 ns (extreme selection criteria) or 8.0 ns (basic selection criteria) [14] are screened to ensure a relatively concentrated energy distribution to the greatest possible extent. Finally, all DEM pixels in the footprint of the ICESat point are averaged using two-dimensional Gaussian weights.

#### 2.1.3. Function Model

According to [10,15], the systematic error of generated DEM can be expressed as a polynomial of image coordinates, as in Equation (2)

(2) $$g_{I}\left(x,y\right)=a_{I}+b_{I}x+c_{I}y$$

where *I* is the index of DEM image, $a_{I}$ is the height offset, and $b_{I}$ and $c_{I}$ are the tilts in ground distance and azimuth, respectively. *x*, *y* are the image coordinates, i.e., ground distance and azimuth with respect to a reference point. The function model can adopt higher-order polynomials, although Runge’s phenomenon, where the model fluctuates violently at the boundary of the adjustment region, may need to be taken into account.

For TPs and GCPs, the observation equations [10,11] are

(3) $$\left[h_{I}^{t}+g_{I}\left(x_{I}^{t},y_{I}^{t}\right)\right]-\left[h_{J}^{t}+g_{J}\left(x_{J}^{t},y_{J}^{t}\right)\right]=l^{t}$$

(4) $$h_{I}^{p}+g_{I}\left(x_{I}^{p},y_{I}^{p}\right)-H_{p}=l^{p}$$

where *t* and *p* are the indexes of TPs and GCPs, *h* is the height extracted from the generated DEM, $H_{p}$ is the height of GCP, and *l* is the residual. Equation (3) is the error equation of a TP chip and represents the elevation difference of different DEMs in the overlapping area. Equation (4) is the error equation of a GCP, and represents the elevation difference between DEM and absolute elevation reference data. Equations (3) and (4) can be further sorted as follows:

(5) $$\begin{array}{cc} l^{t} & =\left[h_{I}^{t}+g_{I}\left(x_{I}^{t},y_{I}^{t}\right)\right]-\left[h_{J}^{t}+g_{J}\left(x_{J}^{t},y_{J}^{t}\right)\right]\\ \; & =g_{I}\left(x_{I}^{t},y_{I}^{t}\right)-g_{J}\left(x_{J}^{t},y_{J}^{t}\right)-\left(h_{J}^{t}-h_{I}^{t}\right)\\ \; & =g_{I}\left(x_{I}^{t},y_{I}^{t}\right)-g_{J}\left(x_{J}^{t},y_{J}^{t}\right)-\Delta h_{t} \end{array}$$

(6) $$\begin{array}{cc} l^{p} & =h_{I}^{p}+g_{I}\left(x_{I}^{p},y_{I}^{p}\right)-H_{p}\\ \; & =g_{I}\left(x_{I}^{p},y_{I}^{p}\right)-\left(H_{p}-h_{I}^{p}\right)\\ \; & =g_{I}\left(x_{I}^{p},y_{I}^{p}\right)-\Delta h_{p} \end{array}$$

For multiple GCPs and TP chips, Equations (5) and (6) can be further sorted into the matrix form as Equation (7):

(7) $$\left[\begin{array}{c} A_{T}\\ A_{P} \end{array}\right]x-\left[\begin{array}{c} \Delta H_{T}\\ \Delta H_{P} \end{array}\right]=\left[\begin{array}{c} L_{T}\\ L_{P} \end{array}\right]$$

where

$$\begin{array}{cc} A_{P} & =diag(A_{P}^{1},\cdots ,A_{P}^{I},\cdots ,A_{P}^{N})\\ A_{P}^{I} & =\left[\begin{array}{ccc} 1 & x_{I}^{1} & y_{I}^{1}\\ 1 & x_{I}^{2} & y_{I}^{2}\\ \vdots & \vdots & \vdots \\ 1 & x_{I}^{p} & y_{I}^{p} \end{array}\right] \end{array}$$

and

$$\begin{array}{cc} \; & A_{T}=\\ \; & \left[\begin{array}{cccccccccc} 1 & x_{1}^{1} & y_{1}^{1} & 0 & \cdots & -1 & -x_{I}^{1} & -y_{I}^{1} & 0 & \cdots \\ \; & \; & & \; & \vdots & \vdots & \; & & \; & \\ 0 & \cdots & 1 & x_{J}^{t} & y_{J}^{t} & \cdots & 0 & -1 & -x_{N}^{t} & -y_{N}^{t} \end{array}\right] \end{array}$$

are the coefficient matrices of TP chips and GCPs, respectively, $x={\left(a_{1},b_{1},c_{1},...,...,a_{N},b_{N},c_{N}\right)}^{T}$ is the vector of unknown parameters, N is the number of generated DEMs, I, J, and K are indexes of generated DEMs, respectively, ΔH is the vector of height differences, and L is the residual vector. The least square solution of Equation (7) is

(8) $$x=A^{†}\Delta H={\left(A_{t}^{T}A_{t}+A_{p}^{T}A_{p}\right)}^{-1}\left(A_{t}^{T}\Delta H_{T}+A_{p}^{T}\Delta H_{p}\right)$$

where $A^{†}={\left(A^{T}A\right)}^{-1}A^{T}$ is the Pseudo-inverse matrix of A.

#### 2.1.4. Shortcomings of the Function Model

The distribution and number of GCPs are the key factors affecting the adjustment results. On the one hand, the distribution of GCPs should be even for the adjustment results to fully reflect the variation in the elevation error with two-dimensional coordinates for ground distance and azimuth. On the other hand, all the generated DEMs involved in the adjustment should contain enough GCPs to prevent the errors of adjacent DEMs from spreading to the whole area through the TPs, leading to deterioration of the adjustment results in the uncontrolled area. In 2011, Ma Jing [5] discussed the influence of the distribution of GCPs on airborne InSAR adjustments. She explained that when the distribution of GCPs is relatively concentrated, the error can be transmitted to the uncontrolled area, resulting in deterioration of the adjustment results.

**Figure 2:**
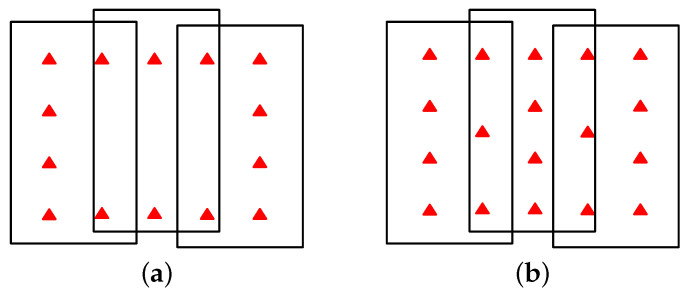
Ideal distribution pattern of GCPs: (**a**) Rectangular shape and (**b**) cross shape; red triangles represent GCPs.

However, GLAH14 data have difficulty meeting the above two requirements, which is not conducive to obtaining reliable and stable adjustment results. Section 3 shows the distribution of GLAH14 data. It can be seen that the distribution pattern of ICESat points is a line along the satellite’s flight trajectory. Conversely, ICESat data does not meet the distribution requirement of GCPs, such as rectangular shape or cross shape in adjustment, as shown in Figure 2. On the other hand, the significant distance between ICESat orbits causes certain images to be adjusted to contain few or no GCPs.

**Figure 3:**
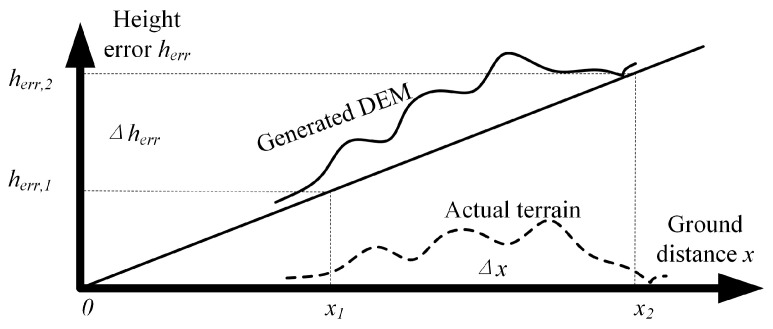
Estimation of range tilt. The dotted line represents the actual terrain, the solid line represents the generated DEM, and the longitudinal axis represents the height difference between the generated DEM and the actual terrain; $x_{1}$ and $x_{2}$ are the ground distance coordinates of two different GCPs, $h_{err,1}$ and $h_{err,2}$ are their systematic elevation errors, and $\Delta h_{err}=h_{err,2}-h_{err,1}$ and $\Delta x=x_{2}-x_{1}$.

Appendix A theoretically analyzes the influence of linear GCP distribution patterns on large-scale DEM block adjustment. First, we demonstrate that all eigenvalues of the coefficient matrix A should be large enough to obtain a stable solution x. Then, we prove that when all GCPs concentrate near a straight line, A has at least a relatively small eigenvalue, which will lead to the instability of x. Here, we take the calculation of the ground tilt *b* as an example, as shown in Figure 3, to provide a visual explanation for the analysis in Appendix A. The estimated value is $\hat{b}\;=\;\Delta h_{err}/\Delta x$ with standard deviation $\sigma_{b}=\sigma_{\Delta h_{err}}/\Delta x$, where $\sigma_{\Delta h}$ is the uncertainty of the height error and Δx is the distance between two different GCPs. It can be seen that $\sigma_{b}$ increases with the decrease of Δx, which means that when the GCPs concentrate at a certain ground distance, that is, Δx is small, the estimated value of $\hat{b}$ will fluctuate greatly. For example, assuming that the swath width of generated DEM is 30 km and $\sigma_{\Delta h_{err}}$ is 0.5 m, and assuming that the uncertainty of ground distance tilt $\hat{b}$ is less than 0.6 m within the width of 30 km, then $\Delta x=\sigma_{\Delta h_{err}}/\sigma_{b}=0.5/\left(0.6/30,000\right)=25$ km, which means that the distance between two GCPs in the direction of ground distance should be greater than 25 km. However, ICESat data cannot always meet this strict condition, where the yellow dots show the location of GLAH14 data). It can be seen that the ICESat data for the adjustment area are concentrated in the middle, around the red solid frame area. The northwest area (i.e., the red dotted frame area) is a large uncontrolled area.

### 2.2. Constraint Slice (CS) and Constraint Conditions

As an essential data source in geoscience the global public DEM have high precision and wide coverage characteristics, as represented by the Shuttle Radar Topography Mission (SRTM) and ALOS World 3D (AW3D30). To solve the problem mentioned in Section 2.1, we tried to introduce the external public global DEM into DEM block adjustment. It should be noted that the vertical accuracy of global public DEM is often far less than that of GLAH14 data and generated DEM. For example, the nominal vertical accuracy of ALOS DEM is 5 m [25] and therefore it cannot be directly added to the adjustment process in the form of GCP.

As discussed in Section 2.1.4, it is difficult to ensure the reliability and stability of adjustment results in the conventional model when GCPs are linearly distributed. Therefore, we tried to overcome this weakness by introducing external terrain data.

Based on the above analysis, we propose a DEM block adjustment method assisted by Constraint Slices (CSs). This method improves the conventional model via two approaches, namely, the concept and extraction approach of Constraint Slices (CSs) and the addition of the elevation variances of CSs to the adjustment optimization model as constraint conditions. The specific process is described as follows.

#### 2.2.1. The Concept and Extraction of Constraint Slices

**Figure 4:**
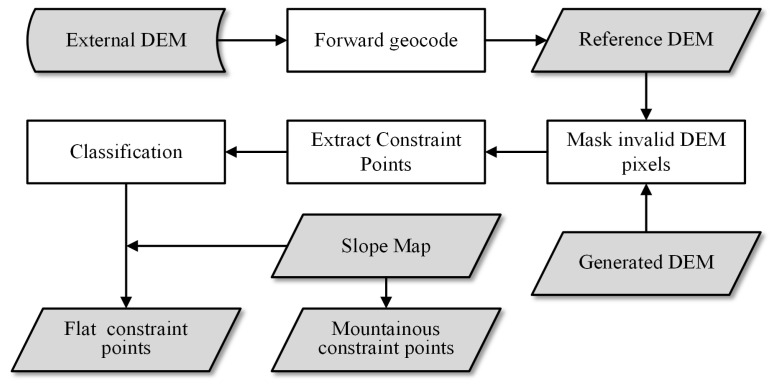
Extraction of Constraint Slices using external public DEM.

CSs are a form of external DEM participating in DEM adjustment. CSs are added to the adjustment optimization model as nonlinear constraint conditions and are extracted as slices, inspired by the TP chips described in Section 2.1.1, which represent the two crucial differences in CSs compared with ordinary GCPs and TPs. A flowchart of the process of extracting CSs is provided in Figure 4. The specific steps are as follows:

i. Geocode the external DEM into the SAR coordinate system (i.e., forward geocoding). This step is usually performed after phase unwrapping because this results in an integer multiple of 2π between the absolute phase and the unwrapped phase [4], which needs to be corrected by external DEM.
ii. Generate the slope map of the reference DEM in the SAR coordinate system. Wang [26] provides a method for generating a slope map using DEM through calculating the gradients in the range and azimuth directions ∂h/∂rg, ∂h/∂az using the Sobel operator and then converting them into a slope map according to Equation (9).
  (9) $$s=\arctan\sqrt{{\left(\frac{\partial h}{\partial rg}\right)}^{2}+{\left(\frac{\partial h}{\partial az}\right)}^{2}}$$
iii. Calculate the elevation differences between the generated and external DEM, and set the pixels with large elevation differences (e.g., greater than 50 m) as invalid pixels, including phase unwrapping error areas and low coherence areas in generated DEMs or elevation anomaly area in external DEMs.
iv. Divide the geocoded DEM into several blocks at an interval of about 1 km. There are two sizes of TP chips in the existing literature, 1 km [10,24] and 500 m [11]. However, because the resolution of the external public DEM is often lower than that of CoSSC data, we assume that a grid can contain more elevation pixels, ensuring the reliability of the CSs. Therefore, inspired by the concept of TP chips and combined with the characteristics of public DEMs, we set the size of the CSs to 1 km. Then, calculate the histogram of each block in the generated and reference DEMs taking the median values of the histograms as their elevations and assign the center coordinates to each block. This part draws on lessons from the extraction method of TPs in Section 2.1.1 in order to avoid the influence of noise elevation outliers.
v. Calculate the average slope of each block and divide the CSs into flat and mountainous areas, as the public DEM often shows different elevation accuracies in mountainous areas and plain areas [21,22]. When the experimental area contains complex terrain, considering only the flat area with the highest accuracy while ignoring the mountainous area will lead to insufficient CSs.

#### 2.2.2. Transformation of Adjustment Model

Taking the relative height accuracy of CSs as nonlinear constraints, we transform the adjustment optimization model into Equation (10):

(10) $$\begin{array}{cc} \min_{x}\; & {∥\mathrm{Ax}-\Delta H∥}_{2}^{2}\\ s.t.\; & Var\left(L_{f}\right)=Var\left(A_{f}x-\Delta H_{f}\right)<\sigma_{f}^{2}\\ \; & Var\left(L_{m}\right)=Var\left(A_{m}x-\Delta H_{m}\right)<\sigma_{m}^{2} \end{array}$$

where *f* and *m* denote CSs of the flat and mountainous region, respectively, $\sigma_{f}$ and $\sigma_{m}$ are the upper limits of the residual standard deviation (which can be empirically obtained from previous studies [18,21,22] or calculated using the GLAH14 data of the experimental area according to the DEM accuracy evaluation method [21,22,27,28]), $A_{f}$ and $A_{m}$ are the coefficient matrices of the CSs, and $\Delta H_{f}$ and $\Delta H_{m}$ are the elevation differences between the CSs and generated DEMs. In Appendix B, we use the Lagrange multiplier method [29] to deduce the solutions of the improved model.

It should be noted that the RMSEs of CSs, i.e., $∥A_{f}x-\Delta H_{f}{∥}_{2}^{2}$ and $∥A_{m}x-\Delta H_{m}{∥}_{2}^{2}$, are not used as constraints here. This is because RMSE represents the absolute height difference between the generated DEM and the external DEM, and using RMSE means taking the external DEM as absolute elevation reference data; in this way, the external DEM is equivalent to the GCPs, as per Equation (4). However, as described in Section 1, the vertical accuracy of the public DEM is usually worse than GLAH14 data, and using RMSEs relative to the external DEM as constraints thus results in lost adjustment accuracy.

## 3. Experiment and Discussion

### 3.1. Experimental Data

**Figure 5:**
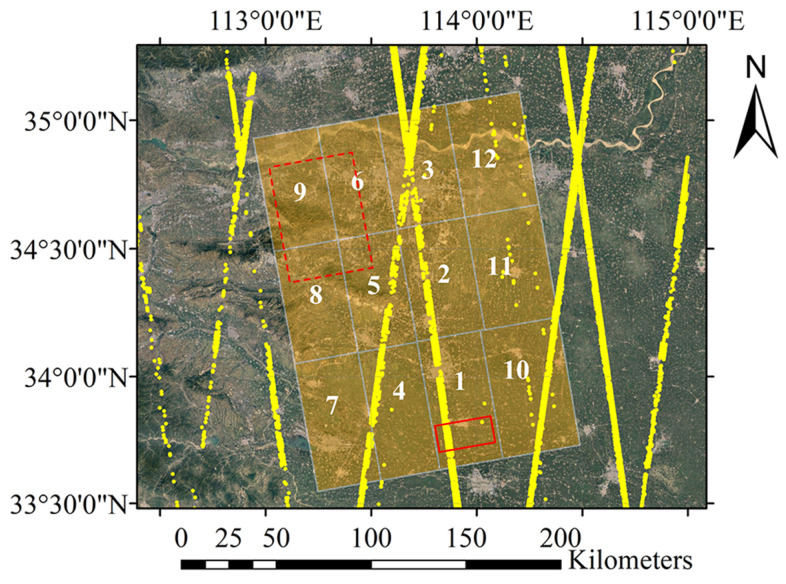
The location of GLAH14 and CoSSC data in the experimental area. The yellow points are GLAH14 data from ICESat. The rectangular windows are the shapes of CoSSC data, and the numbers on them are arranged in the order of their acquisition time.

For this experiment, we selected twelve Co-registered Single-look Slant–range Complex (CoSSC) datasets from TanDEM-X covering Henan Province, China from 2012 to 2013, with a range resolution of about 2.0 m and an azimuth resolution of 1.36 m. The external DEM selected for the experiment was AW3D30, with a resolution of about one arcsec and a nominal vertical accuracy of about 5.0 m. The GCPs used in the experiment consisted of GLAH14 data from 2003 to 2009 selected according to the criterion in [14]. The location relationship between GLAH14 and CoSSC data is shown in Figure 5, in which the serial numbers are arranged according to their acquisition time. The figure shows that the eighth and ninth images do not contain any GCPs, and are thus called the uncontrolled area, while the distribution of GCPs in the other images is uneven. The experimental region contains various geomorphic features with a maximum height difference of about 1400 m. In the southeast is a large plains region, to the northwest are the Songshan Mountains, and to the north lies the Yellow River.

The reference data used were ICESat-2 Land and Vegetation Height data (ATL08), whit a vertical uncertainty of 0.2 m for flat terrain and 2 m for mountainous terrain [30].

### 3.2. Simulated Data

**Figure 6:**
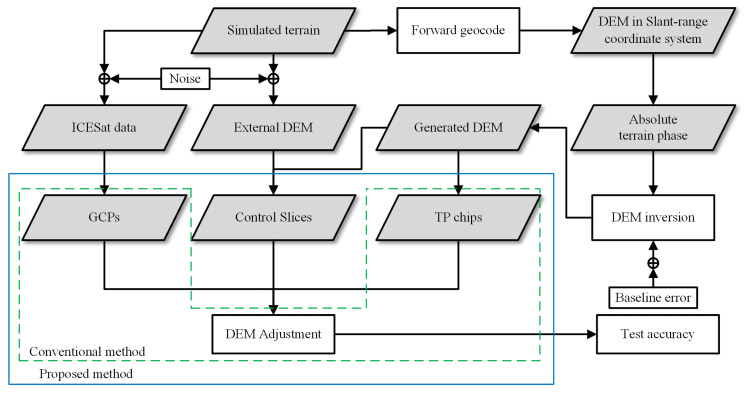
The workflow of the simulated experiment, including all processing steps from simulating external terrain to DEM block adjustment.The green dotted line box represents the traditional adjustment method, and the blue solid line box represents the proposed method.

**Figure 7:**
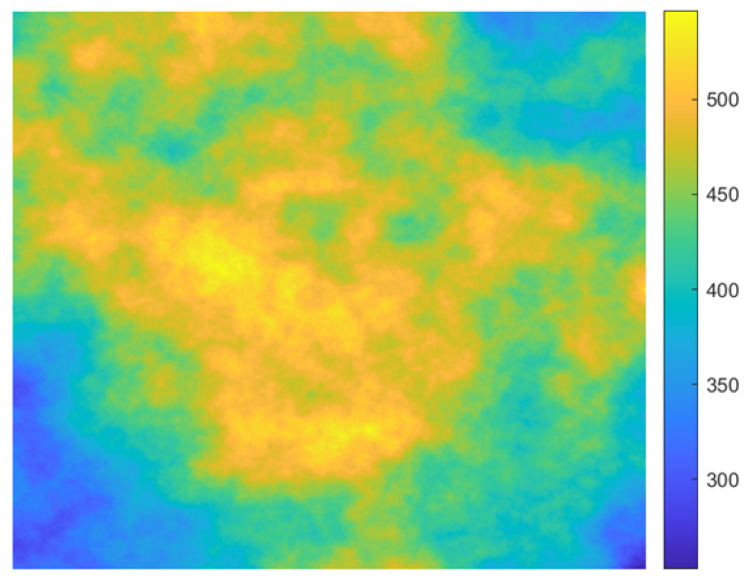
The simulated terrain of the experimental area; the maximum and minimum elevations are 550.0 m and 250.0 m, respectively.

The purpose of this experiment was to illustrate that (1) the traditional methods can lead to inaccurate estimation of adjustment parameters in the case of uneven distribution and insufficient quantity of ICESat data, and (2) the improved method can effectively stabilize the estimated parameters. The central idea of the experiment was to simulate multiple groups of InSAR DEM and compare the performance of the conventional model and proposed model under different baseline errors. The overall flow chart of the simulated experiment is shown as Figure 6, and the specific process is described as follows:

i Simulate the terrain of the experimental area using fractal theory [1], as in Figure 7.
ii Extract ICESat elevation data and generate external DEM. We used the actual geographical coordinates of the ICESat points to extract elevation data from the simulated terrain in step i and added 0.5 m Gaussian noise. Similarly, we added 5.0 m Gaussian noise to the actual terrain as the external DEM.
iii Geocode the simulated terrain. The simulated terrain is geocoded into the slant–range coordinate system using the real satellite orbit information. The geocoded DEM data are used to generate absolute interferometric phase and as the test set of adjustment results.
iv Generate the absolute terrain phase using actual orbit information and the geocoded data from Step iii.
v Carry out InSAR processing, including removing the reference plane phase and generating DEMs. To the master and slave satellite orbit data used in this step were added baseline errors ranging from 0.5 mm to 5.0 mm. In addition, 1.0 m Gaussian noise was added to the generated DEMs. The simulated baseline and random height errors were set based on the actual situation. At present, the baseline accuracy of the bistatic InSAR system is usually on the order of millimeters [2,3]. The published global TanDEM-X DEM has a vertical accuracy of about 1.0 m [31]. Therefore, we chose 0.5 to 5 mm as the baseline error range and 1.0 m as the random noise of the simulated generated DEM.
vi Carry out DEM block adjustment using the conventional and improved method. In the improved method, the CSs are extracted from the external DEM in Step ii. In addition, there is no distinction between mountains and flat land when adding noise in Step ii; thus, the CSs are not distinguished.
vii Accuracy check; checkpoints are evenly selected from the geocoded data from Step iii.

To verify the effectiveness of the proposed method under different degrees of baseline error, we gradually increased the parallel baseline error at intervals of 0.5 mm to generate ten groups of simulation data. We used the root-mean-square error (RMSE) to measure the adjustment accuracy, as follows:

(11) $$\mathrm{RMSE}=\sqrt{\frac{\sum_{i=1}^{N_{ck}}{\left(h_{DEM,i}-h_{check,i}\right)}^{2}}{N_{ck}}}$$

where $h_{DEM}$ and $h_{check}$ are the elevations of the generated DEMs and check points, respectively.

**Figure 8:**
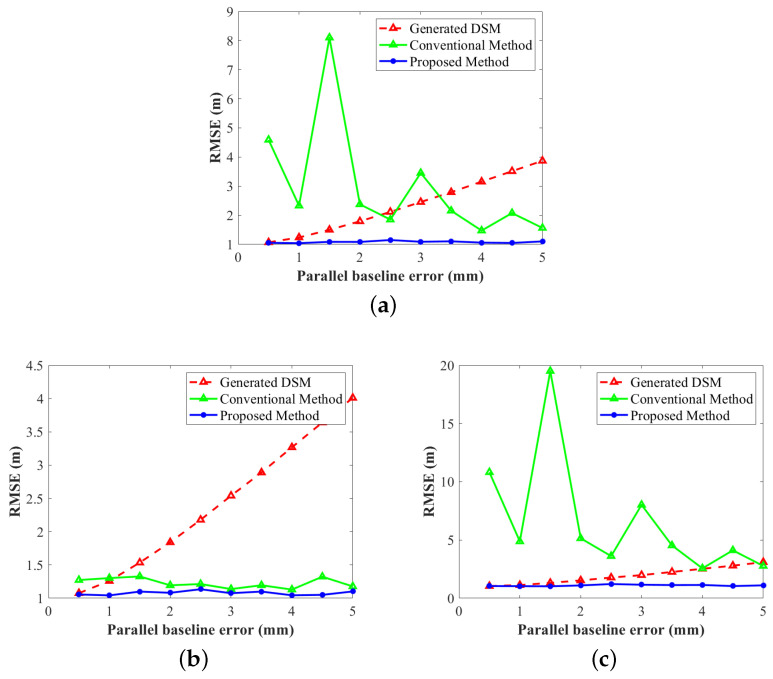
The RMSEs of the simulated experiment results. RMSEs of (**a**) the whole experimental area, (**b**) the controlled area, and (**c**) the uncontrolled area.

The RMSEs of ten sets of data are shown in Figure 8, where Figure 8a–c are the RMSEs of all twelve DEMs, controlled DEMs, and uncontrolled DEMs, respectively. The red, blue, and green lines represent the height RMSEs of the generated DEMs, the adjustment results using the conventional method (7), and the adjustment results using the proposed method (10), respectively. Before adjustment, the elevation errors of the generated DEM are approximately proportional to the baseline errors, which is consistent with Equation (1). Overall, as shown in Figure 8a, the vertical accuracy of traditional methods is unstable and sometimes worse than the original DEM. The RMSEs of the improved method are stable at about 1.0 m, which is very close to the energy of the manually added Gaussian noise in step iii, demonstrating that the improved method nearly eliminates all the systematic errors. In the controlled area, as shown in Figure 8b, both methods show good performance; however, the proposed model is slightly better than the function model. In the uncontrolled area, as in Figure 8c, the adjustment results of traditional methods cannot reflect the actual systematic height error. In addition, the changing trend of RMSEs in Figure 8c is consistent with that in Figure 8a, which confirms that the deterioration of vertical accuracy in uncontrolled areas seriously affects the overall adjustment accuracy. After being constrained by the external DEM, the RMSEs are stable at about 1.0 m.

**Figure 9:**
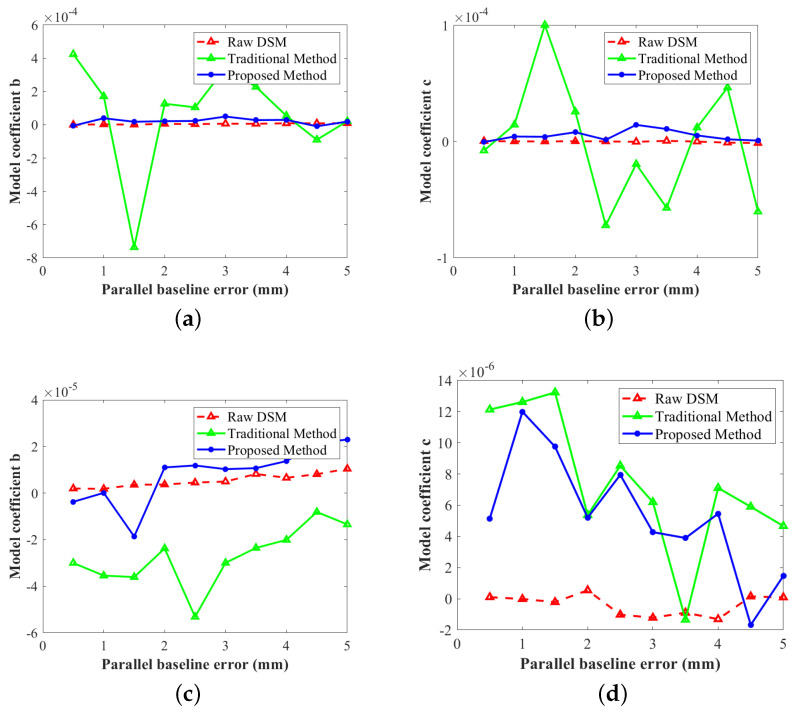
The coefficients of adjustment model: (**a**,**b**) are the range and azimuth tilts of the track containing the seventh to ninth images, respectively, while (**c**,**d**) are the coefficients of the track containing the first to third images.

All in all, the experimental results show that after adding constraint slices the overall adjustment accuracy is significantly improved, which is due to the stability of the adjustment results in the uncontrolled area on the one hand and the improvement of the adjustment accuracy in the controlled area on the other. To further explain these two reasons, we show the range tilt *b* and azimuth tilt *c* of two different orbits in Figure 9. Figure 9a,b show the mean coefficients of the track containing the eighth to ninth CoSSC data (the uncontrolled area), and Figure 9c,d show the mean coefficients of the track containing the first to third CoSSC data (the controlled area). The red, blue, and green lines are the actual values of *b* or *c*, the estimated values of the conventional method, and the estimated values of the proposed method, respectively. As there are no GCPs, the coefficients of the eighth and ninth images fluctuate considerably without constraint slices, as shown in the green lines in Figure 9a,b. The RMSEs of the estimated values are $3.08\times 10^{-4}$ and $5.07\times 10^{-5}$, respectively. However, after introducing constraint slices the adjustment results are stable (shown as blue lines in Figure 9a,b) and the RMSEs of the estimated values are $2.33\times 10^{-5}$ and $6.73\times 10^{-6}$, respectively, which is an order of magnitude lower than the conventional method. The first, second, and third images contain enough GCPs; however, they are distributed unevenly, especially in the ground distance, as shown in Figure 5, which directly results in the offsets of the range and azimuth tilts in Figure 9c,d. The RMSEs of the estimated values using the conventional method (7) are $3.44\times 10^{-5}$ and $8.80\times 10^{-6}$, respectively. After introducing constraint slices, the RMSEs decrease to $1.03\times 10^{-5}$ and $6.89\times 10^{-6}$.

### 3.3. Real Data

**Figure 10:**
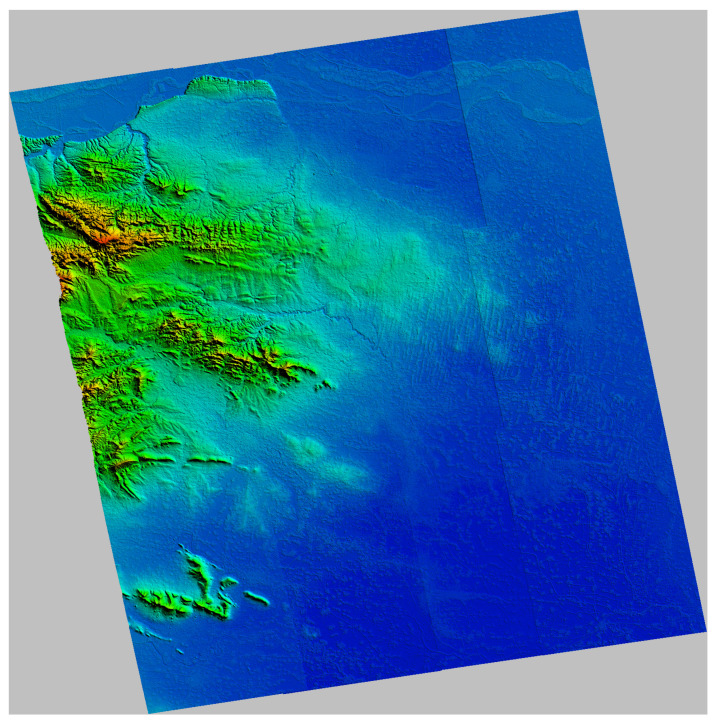
The rendered geocoded map of the generated DEMs; the elevation jumps in the figure are caused by baseline errors.

**Figure 11:**
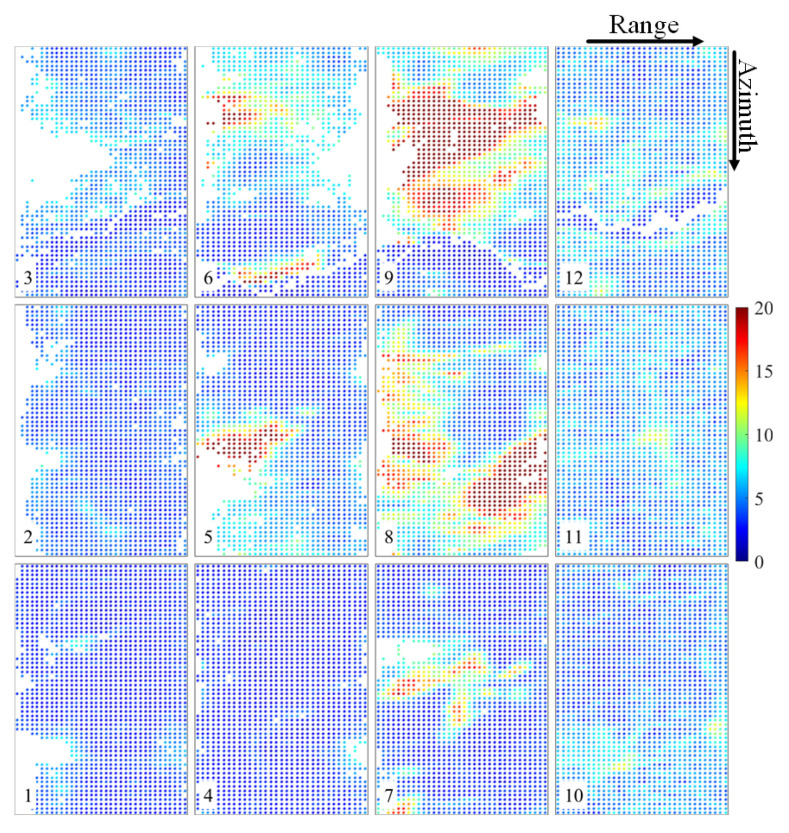
The distribution and slope of the CSs in slant–range coordinates; the color represents the slopes of the CSs. The numbers in the lower left corner are the image number.

**Figure 12:**
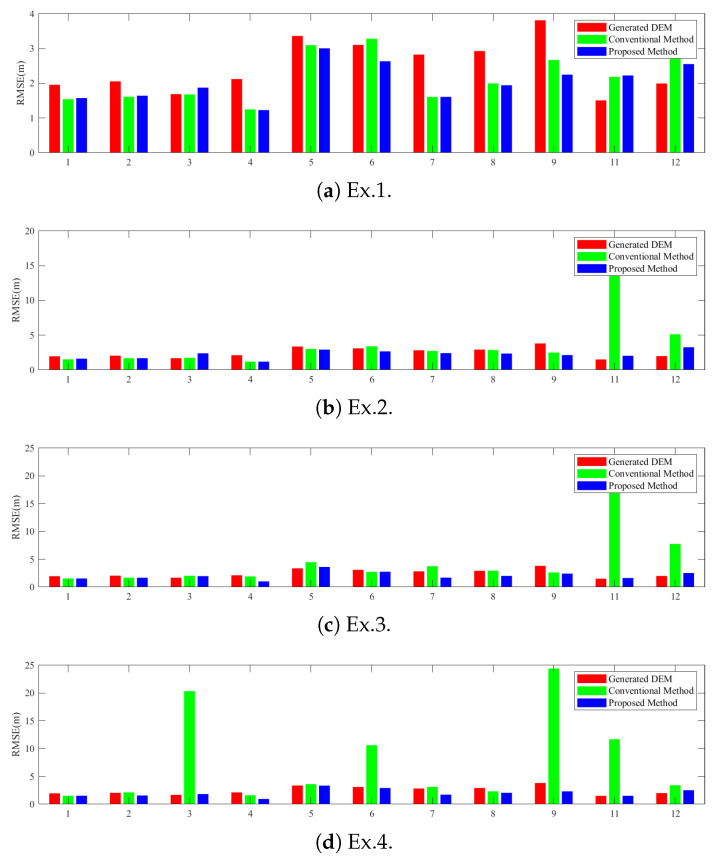
Adjustment results of real data experiment. (**a**–**d**) show the adjustment results under the different sizes of uncontrolled areas. Red, green, and blue bars represent the RMSEs of the generated DEMs, the traditional method, and the proposed method, respectively.

To verify the effectiveness of the proposed method in the actual situation, we changed the size of the uncontrolled area and designed four groups of data experiments. Table 1 shows the data contained in the uncontrolled area in each group of experiments. We generated InSAR DEMs in order to mosaic the geocoded DEMs. Figure 10 displays the geocoded height map of the original DEMs before adjustment. The Yellow River passes through the north of the experimental area, and the phase unwrapping in this area is therefore wrong, resulting in abnormal elevation values. The CSs were extracted from AW3D30 DEM according to the extraction process described in Section 2.2. Figure 11 shows the distribution and slope of the constraint slices in terms of slant–range coordinates. The color represents the slope of CSs. We set the slope threshold to $10^{\circ }$ in order to distinguish mountain CSs and flat CSs. The ICESat-2 ATL08 data were used as check points to verify the accuracy of adjustment results. Unfortunately, the tenth image does not contain any proper check points. Figure 12 shows RMSEs of all images. Table 2 shows RMSEs of the conventional and proposed methods in the controlled, uncontrolled, and general experimental areas.

#### 3.3.1. Performance of Conventional Method in Uncontrolled Areas

The conventional method can maintain a stable adjustment effect when the uncontrolled area is small. Taking Ex.1 as an example, the elevation accuracy of the uncontrolled area before adjustment is 3.08 m and is improved to 2.12 m after adjustment by the conventional method. With the gradual increase of uncontrolled areas, the adjustment results of the conventional method become worse. Taking Ex.4 as an example, prior to adjustment the elevation accuracy of the uncontrolled area is 2.37 m, while after adjustment RMSE deteriorates to 10.41 m.

#### 3.3.2. Performance of Conventional Method in Controlled Areas

The conventional method shows excellent adjustment performance in the controlled area. Taking the first scene data as an example, as shown in Figure 12, the RMSE is 1.96 m before adjustment. After adjustment with the conventional method, the vertical accuracy of the four groups of experiments is stable at about 1.53 m, about 0.4 m better than the original RMSE.

#### 3.3.3. Performance of the Improved Method in Uncontrolled Areas

From Figure 12, the overall adjustment results of the improved method are stable. In Ex.1, the RMSE of the generated DEMs in the uncontrolled area is 3.08 m. The proposed method using CSs brings the RMSE to 1.99 m, and the overall accuracy is improved by about 1.0 m. In Ex.4, before adjustment the vertical accuracy of the uncontrolled area is 2.37 m. After adjustment by the proposed method, the RMSE decreases to 1.96 m, about 8.4 m better than the conventional method. This shows that the improved method can effectively stabilize the adjustment results in the uncontrolled area and improve the vertical accuracy of DEM products.

#### 3.3.4. Performance of the Improved Method in Controlled Areas

In the four experiments, the conventional method improves the vertical accuracies of controlled areas by 0.35 m, 0.20 m, 0.17 m, and 0.43 m, respectively, while the proposed method improves the vertical accuracy by 0.37 m, 0.11 m, 0.20 m, and 0.43 m respectively. Therefore, the performance of the proposed method is similar to the conventional method in the controlled area.

In Table 2, the vertical error of the generated DEM is 2.32 m. The adjustment result of the conventional method is greatly affected by the uncontrolled area. With the increase of the uncontrolled area, the RMSE of the traditional method deteriorates from 1.91 m to 9.78 m. The adjustment results of the proposed method are stable at around 2.0 m, about 0.3 m higher than the original height error. All in all, the data experiment shows that the improved method significantly improves the adjustment results in uncontrolled areas and maintains the adjustment results in controlled areas at the level of the conventional method; thus, the adjustment results tend to be stable and reliable.

**Figure 13:**
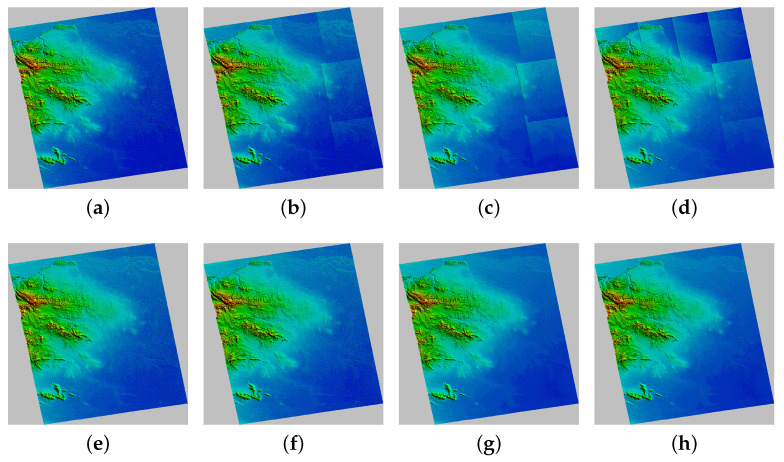
The geocoded map of generated DEMs after adjustment: (**a**–**d**) are adjusted using the conventional method, while (**e**–**h**) are adjusted using the proposed method.

**Figure 14:**
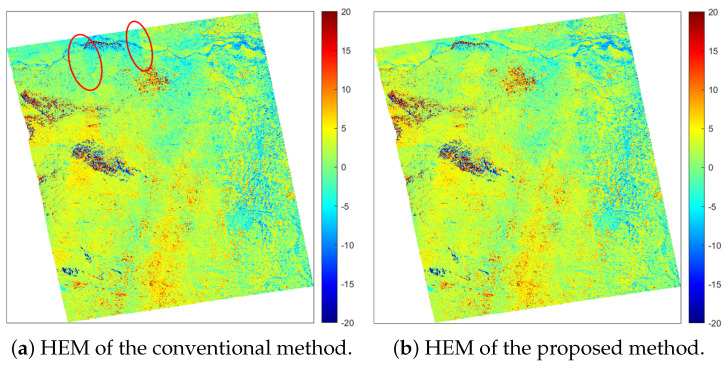
Height error maps of the DEMs adjusted by (**a**) the conventional method and (**b**) the proposed method. The red circles show that the DEMs adjusted by the traditional method have obvious elevation differences in the overlapping area.

Figure 13 shows the geocoded DEM after adjustment where Figure 13a–d are the results of the traditional method and Figure 13e–h are adjusted by the proposed method. It can be seen that the deterioration in the adjustment results in uncontrolled areas leads to significant elevation differences in overlapping areas, resulting in the blocky effect in the geocoded DEM shown in Figure 13b–d. The proposed method can sufficiently suppress the elevation difference, mainly because the public DEM used in the proposed method is seamless. Figure 14 shows the height difference map of Figure 13a,e compared with AW3D30. The suppression effect of the proposed method on elevation difference is reflected significantly in the marked area.

However, our method does has disadvantages. The core idea of our method is to carry out DEM adjustment by introducing global public DEM as auxiliary data. Nevertheless, the measurement time of the external DEM is not consistent with that of the InSAR data. Therefore, elevation changes will inevitably occur when there is a significant time interval between the acquisition of the public DEMs and the CoSSC data, and the resulting CSs then include the systematic elevation error and changes, affecting the final adjustment accuracy. In addition, certain DEMs may have systematic errors, which can affect the accuracy of the CSs. For example, research [25] has pointed out that AW3D30 suffers from the mismatch between adjacent scenes and horizontal and oblique stripping for certain areas. Consequently, to resolve this problem we can introduce multi-source public elevation data, such as SRTM, GDEM, or LiDAR DEM, to develop a robust CS extraction scheme considering data acquisition time and external DEM accuracy. For example, we can assign weights to various external public DEMs according to their acquisition time and obtain the weighted elevation to ensure the reliability of CSs.

## 4. Conclusions

This paper proposed a DEM block adjustment method for spaceborne InSAR using Constraint Slices to solve the deterioration of DEM adjustment results caused by the lack or uneven distribution of GCPs. We selected Constraint Slices from the external public DEM and introduced them into the conventional DEM calibration method in the form of nonlinear constraints. A simulation experiment explained the influence of the lack and uneven distribution of GCPs on the adjustment results and proved that the proposed method could obtain more stable and accurate adjustment results under different degrees of baseline errors. The real data experiment with twelve CoSSC datasets demonstrated that the performance of the proposed method is better than the conventional method in uncontrolled areas and can enhance the elevation consistency of InSAR DEMs in overlapping areas. In the simulated experiment, the overall adjustment accuracy was increased by 0.41 m to 7.01 m. Compared with the conventional method, the adjustment accuracies of the four real data experiments in the uncontrolled area were improved by 0.13 m, 1.02 m, 2.12 m, and 8.45 m, respectively, and the overall adjustment accuracies were improved by 0.02 m, 0.25 m, 1.01 m and 7.86 m, respectively. However, our method still has disadvantages. In the future, it might be possible to introduce multi-source public elevation data in DEM block adjustment to develop a robust CS extraction scheme that considers data acquisition time and external DEM accuracy. 

## Figures and Tables

**Table 1:** The data contained in the uncontrolled area.

Ex. No.	Uncontrolled Area Data	Number of GCPs
Ex. 1	8, 9	324
Ex. 2	7–12	216
Ex. 3	4–12	139
Ex. 4	2–12	34

**Table 2:** RMSEs of real data experiment. The Improvement term represents the improvement in adjustment accuracy when using the proposed method compared to the conventional method.

Ex. No.	Ex. 1			Ex. 2			Ex. 3			Ex. 4		
	RMSE of All / Controlled / Uncontrolled Area (m)											
Before Adjustment	2.32	2.25	3.08	2.32	2.15	2.80	2.32	1.94	2.93	2.32	1.96	2.37
Conventional Method	1.91	1.90	2.12	2.42	1.94	3.54	2.99	1.77	4.51	9.78	1.53	10.41
Proposed Method	1.89	1.88	1.99	2.17	2.04	2.52	1.98	1.74	2.39	1.92	1.53	1.96
Improvement	0.02	0.02	0.13	0.25	-	1.02	1.01	0.03	2.12	7.86	-	8.45

## Data Availability

The data that support the findings of this study are available from the corresponding author upon reasonable request.

## Appendix A. Influence of GCPs Distribution on Eigenvalues of Coefficient Matrix in the Conventional Model

First, we use the Singular Value Decomposition (SVD) method to reveal the relationship between the solution (8) and the eigenvalues of coefficient matrix A. Assuming that the coefficient matrix A is accurate, the uncertainty of observation vector ΔH is δH and the SVD of $A\in R^{m\times n}$ is $A=U\tilde{\Sigma }V^{T}$, where $\tilde{\Sigma }={\left[\Sigma_{1},O\right]}^{T}$ and $\Sigma_{1}=diag\left\{\sigma_{1},\sigma_{2},\cdots ,\sigma_{n}\right\}=diag\left\{\lambda_{1}^{2},\lambda_{2}^{2},\cdots ,\lambda_{n}^{2}\right\}$, σ are the singular values of A and λ represents the eigenvalues of $A^{T}A$. U and **V** are the Unitary matrices. Then, the error of x caused by δH is

(A1) $$\delta x=A^{†}\delta H=V\Sigma U^{T}\delta H$$

where $\Sigma ={\left[\Sigma_{1}^{-1},O\right]}^{T}$ and $\Sigma_{1}^{-1}=diag\left\{1/\lambda_{1}^{2},1/\lambda_{2}^{2},1/\lambda_{3}^{2}\right\}$; (A1) shows that the stability of solution (8) completely depends on the singular values of A. If A has a relatively small eigenvalue, or even an eigenvalue approximately equal to zero, there will be a large error in solution (8).

Assuming that the Jth generated DEM contains n GCPs located on the same satellite trajectory and that the geographical coordinates of each GCP are $\left(x_{i},y_{i}\right)$, then the coefficient matrix is

(A2) $$A_{J}=\left[\begin{array}{ccc} 1 & x_{1} & y_{1}\\ 1 & x_{2} & y_{2}\\ \vdots & \vdots & \vdots \\ 1 & x_{n} & y_{n} \end{array}\right]$$

and

(A3) $$A_{J}^{T}A_{J}=\left[\begin{array}{ccc} n & \sum_{i}x_{i} & \sum_{i}y_{i}\\ \sum_{i}x_{i} & \sum_{i}x_{i}^{2} & \sum_{i}x_{i}y_{i}\\ \sum_{i}y_{i} & \sum_{i}x_{i}y_{i} & \sum_{i}y_{i}^{2} \end{array}\right]$$

According to the distribution characteristics of ICESat points, it can be assumed that the geographical coordinates $x_{i}$ and $y_{i}$ of GCPs are approximately distributed on the same straight line in each generated DEM, i.e.,

(A4) $$y_{i}=kx_{i}+b+\varepsilon_{i}=\tilde{y_{i}}+\varepsilon_{i}$$

where *k* and *b* are the slope and intercept of the fitted line, respectively, $\tilde{y_{i}}$ is the fitting value corresponding to $x_{i}$, and $\varepsilon_{i}$ is the offset between real coordinates and the fitted line. In fact, as shown by the yellow points in Figure 5, $\varepsilon_{i}$ is tiny relative to $x_{i}$ and $y_{i}$ because of the stable satellite orbit. Replace $y_{i}$ with Equation (A4), and Equation (A3) can be expanded as Equation (A5), where $\delta B_{J}$ is called perturbation of $B_{J}$ in perturbation theory [32]. It can be proved that $B_{J}$ is rank-deficient, that is, $B_{J}$ has at least one zero eigenvalue.

(A5) $$\begin{array}{cc} A_{J}^{T}A_{J} & =\left[\begin{array}{ccc} n & \sum_{i}x_{i} & \sum_{i}\tilde{y_{i}}+\sum_{i}\varepsilon_{i}\\ \sum_{i}x_{i} & \sum_{i}x_{i}^{2} & \sum_{i}x_{i}\tilde{y_{i}}+\sum_{i}x_{i}\varepsilon_{i}\\ \sum_{i}\tilde{y_{i}}+\sum_{i}\varepsilon_{i} & \sum_{i}x_{i}\tilde{y_{i}}+\sum_{i}x_{i}\varepsilon_{i} & \sum_{i}{\tilde{y_{i}}}^{2}+2\sum_{i}\varepsilon_{i}\tilde{y_{i}}+\sum_{i}\varepsilon_{i}^{2} \end{array}\right]\\ \; & =\left[\begin{array}{ccc} n & \sum_{i}x_{i} & \sum_{i}\tilde{y_{i}}\\ \sum_{i}x_{i} & \sum_{i}x_{i}^{2} & \sum_{i}x_{i}\tilde{y_{i}}\\ \sum_{i}\tilde{y_{i}} & \sum_{i}x_{i}\tilde{y_{i}} & \sum_{i}{\tilde{y_{i}}}^{2} \end{array}\right]+\left[\begin{array}{ccc} 0 & 0 & \sum_{i}\varepsilon_{i}\\ 0 & 0 & \sum_{i}x_{i}\varepsilon_{i}\\ \sum_{i}\varepsilon_{i} & \sum_{i}x_{i}\varepsilon_{i} & 2\sum_{i}\varepsilon_{i}\tilde{y_{i}}+\sum_{i}\varepsilon_{i}^{2} \end{array}\right]\\ \; & =B_{J}+\delta B_{J} \end{array}$$

Suppose the eigenvalues of $A_{J}^{T}A_{J}$ are $\left\{\sigma_{J,1}^{*},\sigma_{J,2}^{*},\sigma_{J,3}^{*}\right\}$, $\sigma_{J,1}^{*}\geq \sigma_{J,2}^{*}\geq \sigma_{J,3}^{*}\geq 0$ and the eigenvalues of $B_{J}$ are $\left\{\sigma_{J,1},\sigma_{J,2},\sigma_{J,3}\right\}$, $\sigma_{J,1}\geq \sigma_{J,2}\geq \sigma_{J,3}=0$; then,

(A6) $$\sigma_{J,1}^{*}+\sigma_{J,2}^{*}+\sigma_{J,3}^{*}=\mathrm{tr}\left(A_{J}^{T}A_{J}\right)=n+\sum_{i}x_{i}^{2}+\sum_{i}{\tilde{y_{i}}}^{2}+2\sum_{i}\varepsilon_{i}\tilde{y_{i}}+\sum_{i}\varepsilon_{i}^{2}$$

where $\mathrm{tr}(A_{J}^{T}A_{J})$ is the trace of $A_{J}^{T}A_{J}$. Then, according to the Weyl monotonicity theorem [33,34],

(A7) $$\sigma_{J,3}^{*}\leq {∥\delta B_{J}∥}_{spec}=\lambda_{max}\left(\delta B_{J}\right)$$

where $∥\delta B_{J}{∥}_{spec}$ is the spectrum norm of $B_{J}$, that is, the maximum eigenvalue of $B_{J}$, as Equation (A8):

(A8) $$\begin{array}{cc} \lambda_{max}\left(\delta B_{J}\right) & =\frac{2\sum_{i}\varepsilon_{i}\tilde{y_{i}}+\sum_{i}\varepsilon_{i}^{2}}{2}+\frac{\sqrt{4{\left(\sum_{i}\varepsilon_{i}\right)}^{2}+4{\left(\sum_{i}x_{i}\varepsilon_{i}\right)}^{2}+{\left(2\sum_{i}\varepsilon_{i}\tilde{y_{i}}+\sum_{i}\varepsilon_{i}^{2}\right)}^{2}}}{2}\\ \; & <|\sum_{i}\varepsilon_{i}\left|+\right|\sum_{i}x_{i}\varepsilon_{i}|+|2\sum_{i}\varepsilon_{i}\tilde{y_{i}}+\sum_{i}\varepsilon_{i}^{2}| \end{array}$$

In order to make the analysis more clear, we assume that ε is a random variable with zero expectation and independent of the geographical coordinates of ICESat points (which, in fact, it is). Then, Equations (A6) and (A8) are arranged into statistical form as

(A9) $$\begin{array}{cc} \; & \sigma_{J,1}^{*}+\sigma_{J,2}^{*}+\sigma_{J,3}^{*}\\ \; & =n+\sum_{i}x_{i}^{2}+\sum_{i}{\tilde{y_{i}}}^{2}+2\sum_{i}\varepsilon_{i}\tilde{y_{i}}+\sum_{i}\varepsilon_{i}^{2}\\ \; & =n+\sum_{i}x_{i}^{2}+\sum_{i}{\tilde{y_{i}}}^{2}+2nE\left(\varepsilon \tilde{y}\right)+nE\left(\varepsilon^{2}\right)\\ \; & =n+\sum_{i}x_{i}^{2}+\sum_{i}{\tilde{y_{i}}}^{2}+nE\left(\varepsilon^{2}\right) \end{array}$$

(A10) $$\begin{array}{cc} \sigma_{J,3}^{*} & \leq \lambda_{max}\left(\delta B_{J}\right)\\ \; & <|\sum_{i}\varepsilon_{i}\left|+\right|\sum_{i}x_{i}\varepsilon_{i}|+|2\sum_{i}\varepsilon_{i}\tilde{y_{i}}+\sum_{i}\varepsilon_{i}^{2}|\\ \; & =n|E\left(\varepsilon \right)|+n|E\left(x\varepsilon \right)|+n|2E\left(\varepsilon \tilde{y}\right)+E\left(\varepsilon^{2}\right)|\\ \; & =nE(\varepsilon^{2}) \end{array}$$

where E(·) is an expectation operator. Then, combining Equations (A9) and (A10), we can obtain

(A11) $$\frac{\sigma_{J,3}^{*}}{\sigma_{J,1}^{*}+\sigma_{J,2}^{*}}\leq \frac{nE(\varepsilon^{2})}{n+\sum_{i}x_{i}^{2}+\sum_{i}{\tilde{y_{i}}}^{2}}=\frac{E(\varepsilon^{2})}{1+\frac{\sum_{i}x_{i}^{2}}{n}+\frac{\sum_{i}{\tilde{y_{i}}}^{2}}{n}}\ll 1$$

Equation (A11) shows that the third eigenvalue $\sigma_{J,3}^{*}$ is particularly small compared with the sum of the other two eigenvalues of $A_{J}^{T}A_{J}$.

## Appendix B. Solution of the Constraint Model

The Lagrange function corresponding to the constraint model Equation (10) is

(A12) $$\begin{array}{cc} L(x,\lambda ,\mu ) & ={∥\mathrm{Ax}-\Delta H∥}_{2}^{2}+\lambda \left[Var\left(A_{f}x-\Delta H_{f}\right)-\sigma_{f}^{2}\right]+\mu \left[Var\left(A_{m}x-\Delta H_{m}\right)-\sigma_{m}^{2}\right]\\ \; & ,\lambda ,\mu \geq 0 \end{array}$$

where $A_{f}\in R^{n_{1}\times 3}$ and $A_{m}\in R^{n_{2}\times 3}$$n_{1}$ and $n_{2}$ represent the numbers of flat and mountain CSs, respectively. Var(·) is the variance operator and can be expanded as

(A13) $$\begin{array}{cc} \; & Var(A_{f}x-\Delta H_{f})\\ \; & ={\left(A_{f}x-\Delta H_{f}-m_{f}I_{n_{1}\times 1}\right)}^{T}\left(A_{f}x-\Delta H_{f}-m_{f}I_{n_{1}\times 1}\right)\\ \; & ={\left(A_{f}x-\Delta H_{f}\right)}^{T}\left(A_{f}x-\Delta H_{f}\right)\\ \; & -\frac{1}{n_{1}}{\left(A_{f}x-\Delta H_{f}\right)}^{T}1_{n_{1}\times n_{1}}\left(A_{f}x-\Delta H_{f}\right) \end{array}$$

where $I_{n\times 1}$ is an all-1 vector, $1_{n\times n}$ is an all-1 matrix, and $m_{f}={\left(A_{f}x-\Delta H_{f}\right)}^{T}I_{n_{1}\times 1}$ is the mean value of vector $A_{f}x-\Delta H_{f}$. $Var(A_{m}x-\Delta H_{m})$ can be expanded as Equation (A13). Then, the gradient function of Equation (A12) with respect to x is

(A14) $$\begin{array}{cc} \; & \nabla_{x}L\left(x,\lambda ,\mu \right)=A^{T}\left(Ax-\Delta H\right)\\ \; & +\lambda \left[A_{f}^{T}\left(A_{f}x-\Delta H_{f}\right)-\frac{1}{n_{1}}A_{f}^{T}1_{n_{1}\times n_{1}}\left(A_{f}x-\Delta H_{f}\right)\right]\\ \; & +\mu \left[A_{m}^{T}\left(A_{m}x-\Delta H_{m}\right)-\frac{1}{n_{2}}A_{m}^{T}1_{n_{2}\times n_{2}}\left(A_{m}x-\Delta H_{m}\right)\right]\\ \; & =A^{T}\left(Ax-\Delta H\right)+\lambda {\tilde{A}}_{f}^{T}\left(A_{f}x-\Delta H_{f}\right)\\ \; & +\mu {\tilde{A}}_{m}^{T}\left(A_{m}x-\Delta H_{m}\right) \end{array}$$

For simplicity, we replace $\left(E_{n_{1}}-\frac{1}{n_{1}}I_{n_{1}\times n_{1}}\right)A_{f}$ and $\left(E_{n_{2}}-\frac{1}{n_{2}}I_{n_{2}\times n_{2}}\right)A_{m}$ in Equation (A14) with ${\tilde{A}}_{f}$ and ${\tilde{A}}_{m}$, respectively; E is an unit matrix.

Finally, we list the Karush–Kuhn–Tucker conditions (KKT conditions) [29], as follows:

(A15) $$\left\{\begin{array}{cc} \; & \nabla_{x}L=0\\ \; & \nabla_{\lambda }L=Var\left(A_{f}x-\Delta H_{f}\right)-\sigma_{f}^{2}\leq 0\\ \; & \nabla_{\mu }L=Var\left(A_{m}x-\Delta H_{m}\right)-\sigma_{m}^{2}\leq 0\\ \; & \lambda ,\mu \geq 0\\ \; & \lambda \nabla_{\lambda }L,\mu \nabla_{\mu }L=0 \end{array}\right.$$

which can be solved iteratively by classical constrained optimization algorithms such as the Interior Point algorithm [35]. The iteration result of the kth step is

(A16) $$\begin{array}{cc} x= & {\left(A^{T}A+\lambda_{k}{\tilde{A}}_{f}^{T}A_{f}+\mu_{k}{\tilde{A}}_{m}^{T}A_{m}\right)}^{-1}\left(A^{T}\Delta H+\lambda_{k}{\tilde{A}}_{f}^{T}\Delta H_{f}+\mu_{k}{\tilde{A}}_{m}^{T}\Delta H_{m}\right)\\ = & {\left(A_{t}^{T}A_{t}+A_{p}^{T}A_{p}+\lambda_{k}{\tilde{A}}_{f}^{T}A_{f}+\mu_{k}{\tilde{A}}_{m}^{T}A_{m}\right)}^{-1}\left(A_{t}^{T}\Delta H_{t}+A_{p}^{T}\Delta H_{p}+\lambda_{k}{\tilde{A}}_{f}^{T}\Delta H_{f}+\mu_{k}{\tilde{A}}_{m}^{T}\Delta H_{m}\right) \end{array}$$
